# Imeglimin attenuates liver fibrosis by inhibiting vesicular ATP release from hepatic stellate cells

**DOI:** 10.1002/1873-3468.70166

**Published:** 2025-09-15

**Authors:** Seiji Nomura, Lixiang Wang, Nao Hasuzawa, Ayako Nagayama, Sawako Moriyama, Kenji Ashida, Yoshinori Moriyama, Masatoshi Nomura, Ken Yamamoto

**Affiliations:** ^1^ Department of Medical Biochemistry Kurume University School of Medicine Japan; ^2^ Division of Endocrinology and Metabolism, Department of Internal Medicine Kurume University School of Medicine Japan

**Keywords:** ATP, hepatic stellate cells, imeglimin, liver fibrosis, purinergic signaling, vesicular ATP release, VNUT

## Abstract

The protective effects of imeglimin, a recently approved antidiabetic agent, against liver fibrosis have not been previously evaluated. In this study, we demonstrated that 8‐week administration of imeglimin attenuated immune cell infiltration and reduced collagen deposition, improving fibrosis stage in a thioacetamide‐induced murine model. Further analyses focusing on hepatic stellate cells (HSCs), the primary effector cells in fibrogenesis, revealed decreased expression of α‐smooth muscle actin and desmin, markers of HSC activation. Mechanistically, a clinically relevant low concentration (10 μm) of imeglimin reduced intracellular vesicular ATP accumulation and subsequently suppressed ATP release from HSCs *in vitro*. These findings suggest that imeglimin may exert anti‐inflammatory and antifibrotic effects by inhibiting vesicular ATP release and ATP‐mediated purinergic signaling.

**Impact statement**

At clinically relevant doses, imeglimin inhibits vesicular ATP release from hepatic stellate cells, reducing inflammatory infiltration and fibrotic collagen accumulation. These findings support its evaluation as a combined metabolic and antifibrotic therapy for MASLD and other chronic liver conditions.

## Graphical abstract

Imeglimin, at clinically relevant concentrations, inhibits vesicular ATP accumulation and release from hepatic stellate cells, thereby attenuating purinergic signaling and reducing fibrogenic activation. This mechanism reveals a newly identified antifibrotic action of imeglimin beyond glycemic control.

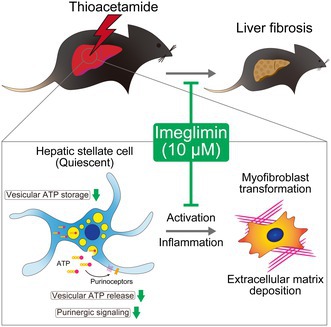

## Abbreviations

**CLD**, chronic liver disease

**HSC**, hepatic stellate cell

**IMG**, imeglimin

**MASH**, metabolic dysfunction‐associated steatohepatitis

**MASLD**, metabolic dysfunction‐associated steatotic liver disease

**TAA**, thioacetamide

**VNUT**, vesicular nucleotide transporter

Chronic liver disease (CLD) and cirrhosis represent a significant global health burden, affecting approximately 1.5 billion individuals worldwide, with over 2 million deaths annually attributable to cirrhosis [1]. The principal etiologies of CLD include chronic viral hepatitis caused by hepatitis B virus (HBV) and hepatitis C virus (HCV), alcohol‐related liver disease (ALD), and metabolic dysfunction‐associated steatotic liver disease (MASLD), which is commonly associated with diabetes and obesity. The global prevalence of viral hepatitis has declined due to the widespread implementation of HBV vaccination programs and the availability of effective antiviral therapies for HBV and HCV [2]. In contrast, the prevalence of MASLD and its progressive form, metabolic dysfunction‐associated steatohepatitis (MASH), is increasing and has become a major contributor to cirrhosis and hepatocellular carcinoma (HCC) [3], in parallel with the global rise in obesity and type 2 diabetes mellitus (T2DM) [4]. Consequently, the overall burden of CLD and cirrhosis remains substantial and is projected to continue increasing [1].

Chronic inflammation plays a central role in the pathogenesis of CLD. In particular, activation of hepatic stellate cells (HSCs), which transdifferentiate into fibrogenic myofibroblasts, is a pivotal event that leads to excessive extracellular matrix (ECM) deposition and the development of liver fibrosis [5]. Persistent hepatic inflammation drives fibrogenesis and significantly increases the risk of progression to cirrhosis and HCC [6]. Despite the clinical importance of liver fibrosis, the development of antifibrotic therapies targeting HSCs and ECM deposition has proven to be highly challenging, and no effective pharmacological agent is currently available [7]. This represents a critical and unresolved therapeutic need.

Imeglimin is a novel oral antidiabetic agent approved for the treatment of T2DM. It exerts its glucose‐lowering effects through multiple mechanisms, including the enhancement of glucose‐stimulated insulin secretion in pancreatic β‐cells and the improvement of insulin sensitivity in peripheral tissues, particularly the liver and skeletal muscle [8]. In the liver, imeglimin reduces hepatic gluconeogenesis and enhances insulin signaling, while in skeletal muscle, it promotes glucose uptake and improves mitochondrial function. These combined effects contribute to the maintenance of glucose homeostasis.

In addition to its metabolic effects, emerging preclinical evidence suggests that imeglimin possesses anti‐inflammatory properties. It has been shown to inhibit the activation of the NOD‐like receptor family pyrin domain‐containing 3 (NLRP3) inflammasome and to downregulate the expression of proinflammatory cytokines, including interleukin (IL)‐1β, tumor necrosis factor‐α (TNF‐α), and IL‐6, in peripheral blood mononuclear cells [9]. Furthermore, *in vitro* studies using endothelial cells have demonstrated that imeglimin attenuates hyperglycemia‐induced inflammatory responses without compromising cellular energy status [10], suggesting a potential for systemic anti‐inflammatory effects independent of glycemic control.

Given the overlapping metabolic and inflammatory components of T2DM and MASLD, therapeutic agents capable of modulating both processes are of considerable interest. Imeglimin, owing to its dual glucose‐lowering and anti‐inflammatory properties, may hold promise as a candidate for attenuating hepatic inflammation and fibrosis. However, while its metabolic effects on the liver are increasingly well‐characterized, its potential impact on hepatic inflammatory and fibrotic pathways remains insufficiently understood. To address this gap, we conducted an *in vivo* study to investigate the molecular effects of imeglimin in the liver, with a particular focus on its putative anti‐inflammatory and antifibrotic properties.

## Materials and methods

### Animal experiments

C57BL/6 wild‐type male mice (Charles River Laboratories Japan, Inc., Yokohama, Kanagawa, Japan) were housed under a 12‐h light/dark cycle and were provided *ad libitum* access to a standard chow diet (5.4% fat, CRF‐1; Orient Yeast Co., Ltd., Tokyo, Japan). Thioacetamide (TAA, Fujifilm Wako Pure Chemical Corporation, Osaka, Japan) was dissolved in 0.9% (w/v) saline for intraperitoneal (i.p.) injection. Imeglimin (IMG, Sumitomo Pharma Co., Ltd., Osaka, Japan) was prepared in 0.9% (w/v) saline for oral administration (per os, p.o.) using an oral gavage. Eight‐week‐old mice were randomly divided into three groups and treated over a period of nine consecutive weeks as follows:

1. Control group (*n* = 6): Mice received vehicle p.o. three times per week following i.p. injection of saline.
2. TAA group (*n* = 7): Mice received vehicle p.o. three times per week following i.p. injection of TAA (50 mg·kg^−1^) to induce liver fibrosis.
3. TAA + IMG group (*n* = 7): Mice received vehicle p.o. during the first week, followed by IMG (200 mg·kg^−1^, p.o.) for the subsequent 8 weeks, three times per week. IMG administration was performed following TAA (50 mg·kg^−1^, i.p.) injection.

All mice were euthanized 1 h after the final administration of IMG or vehicle, in accordance with their respective group assignments. Euthanasia was performed following the collection of liver and blood samples under isoflurane anesthesia (3 L·min^−1^), by cervical dislocation. Biochemical parameters and complete blood count in mouse blood were analyzed using the Vetscan VS2 chemistry analyzer and Vetscan HM5 hematology analyzer, respectively (Zoetis Inc., Parsippany, NJ, USA). The concentrations of inflammatory cytokines—IL‐1β (Cat. No. 560232), TNF (Cat. No. 558299), IL‐6 (Cat. No. 558301), and Monocyte chemoattractant protein‐1 (MCP‐1; Cat. No. 558342)—in mouse plasma and liver tissue homogenates were quantified with Cytometric Bead Array Flex Sets (BD Biosciences, San Jose, CA, USA), followed by NovoCyte Flow cytometer (ACEA Biosciences, Inc., San Diego, CA, USA) and NovoExpress software. All experiments were carried out using male mice, in accordance with institutional guidelines and were approved by the Ethics Committee of the Kurume University School of Medicine (approval number: 2024‐142).

### Histology

Liver tissue samples were fixed in 4% paraformaldehyde, embedded in paraffin, sectioned, and stained with hematoxylin and eosin (H&E) or Picrosirius Red (PSR). Fibrosis staging was assessed by a blinded investigator using a standardized METAVIR scoring system ranging from F0 to F4 [11]. Immunohistochemical staining was performed on paraffin‐embedded tissue sections using a monoclonal antibody against α‐smooth muscle actin (α‐SMA, 1 500 dilution, A5228; Sigma‐Aldrich, St. Louis, MO, USA), F4/80 (1 : 500, MCA497GA; Bio‐Rad AbD Serotec, Kidlington, Oxford, UK), and a polyclonal antibody against desmin (1:200, 16520‐1; Proteintech Group, Inc., Rosemont, IL, USA). Donkey anti‐rabbit IgG conjugated with Alexa Fluor 488 (1 : 500 dilution, ab150073; Abcam plc, Cambridge, UK) was used as a secondary antibody, followed by nuclear counterstaining with NucBlue (Hoechst 33342, R37605; Thermo Fisher Scientific, Waltham, MA, USA). Alternatively, horseradish peroxidase (HRP)‐conjugated mouse anti‐rabbit secondary antibody (7074; Cell Signaling Technology, Danvers, MA, USA) and 3,3′‐diaminobenzidine (DAB) were used for detection. Stained sections were examined using a BZ‐X710 microscope (Keyence Corporation, Osaka, Japan). The areas of immune cell infiltration were measured, and the number of F4/80‐positive cells was counted in five high‐power fields per section. Quantification of α‐SMA, desmin, and PSR‐positive area was performed using Fiji (ImageJ, version 1.54f).

### RT‐qPCR

Total RNA was isolated from the mouse liver using TRIzol Reagent (Invitrogen (Thermo Fisher Scientific), Carlsbad, CA, USA). Reverse transcription with 1 μg of RNA was conducted using the QuantiTect Reverse Transcription Kit (Qiagen GmbH, Hilden, Germany). Quantitative real‐time PCR (RT‐qPCR) was used to determine the relative expression levels of mRNA. The assays were performed with SYBR Premix Ex Taq II (Takara Bio Inc., Kusatsu, Shiga, Japan) on the Applied Biosystems StepOne Plus Real‐Time PCR system. The primer sequences of the selected genes are provided below. Results were normalized to the expression of 18S ribosomal RNA (*18s*) and are shown as the fold change relative to gene expression in the control.

*Il1b*

Forward: 5′‐ TGTGAAATGCCACCTTTTGA ‐ 3′.

Reverse: 5′‐ GGTCAAAGGTTTGGAAGCAG ‐ 3′.

*Tnfa*

Forward: 5′‐ CCACCACGCTCTTCTGTCTA ‐ 3′.

Reverse: 5′‐ AGGGTCTGGGCCATAGAACT ‐ 3′.

*Il6*

Forward: 5′‐ TCCAGTTGCCTTCTTGGGAC ‐ 3′.

Reverse: 5′‐ AGTCTCCTCTCCGGACTTGT ‐ 3′.

*Mcp1*

Forward: 5′‐ GCAGTTAACGCCCCACTCA ‐ 3′.

Reverse: 5′‐ CCAGCCTACTCATTGGGATCA ‐ 3′.

*Timp1*

Forward: 5′‐ GTGGGAAATGCCGCAGAT ‐ 3′.

Reverse: 5′‐ GGGCATATCCACAGAGGCTTT ‐ 3′.

*Sma*

Forward: 5′‐ ATCCGATAGAACACGGCATC ‐ 3′.

Reverse: 5′‐ GTTCAGTGGTGCCTCTGTCA ‐ 3′.

*Col1a1*

Forward: 5′‐ CCTGGACAGCCTGGACTTC ‐ 3′.

Reverse: 5′‐ CCATAGGACATCTGGGAAGC ‐ 3′.

*18s*

Forward: 5′‐ ‐AAACGGCTACCACATCCAAG ‐ 3′.

Reverse: 5′‐ CCTCCAATGGATCCTCGTTA ‐ 3′.

### Western blotting

Mouse liver tissues were homogenized in a lysis buffer containing 20 mm Tris–HCl (pH 7.6), 150 mm NaCl, 2 mm ethylenediaminetetraacetic acid (EDTA), and 0.5% NP‐40, supplemented with protease and phosphatase inhibitor tablets (Roche Diagnostics GmbH (formerly Roche Applied Science), Mannheim, Germany). Protein concentrations were determined using the bicinchoninic acid protein assay kit (Thermo Fisher Scientific). Equal amounts of protein (10 μg per lane) were mixed with Laemmli sample buffer (Bio‐Rad Laboratories, Inc., Hercules, CA, USA), boiled at 95 °C for 5 min, and subjected to sodium dodecyl sulfate–polyacrylamide gel electrophoresis. Proteins were then transferred onto polyvinylidene difluoride membranes. After blocking, membranes were incubated overnight at 4 °C with the following primary antibodies: anti‐α‐SMA (A5228; Sigma‐Aldrich), anti‐Collagen I (COL1A1, NB600‐408; Novus Biologicals, Centennial, CO, USA), anti‐Transforming growth factor‐beta 1 (TGF‐β1, ab215715; Abcam), anti‐Matrix metalloproteinase‐1 (MMP‐1, 54376S; Cell Signaling Technology), and anti‐β‐Actin (4970S; Cell Signaling Technology). Subsequently, membranes were incubated for 1 h at room temperature with horseradish peroxidase (HRP)‐conjugated secondary antibodies (7074P2; Cell Signaling Technology). Visualization of protein bands was achieved using Ez WestLumi plus (ATTO Corporation, Tokyo, Japan), and signal detection was performed using a chemiluminescence‐based western blotting detection system (LAS 4000; Fujifilm Corporation, Tokyo, Japan). The relative band intensities were quantified using Fiji with a custom macro plugin (Kenji Ohgane, Hiromasa Yoshioka. Quantification of Gel Bands by an imagej Macro, Band/Peak Quantification Tool. protocols.io. https://doi.org/10.17504/protocols.io.7vghn3w).

### Cell culture

The human hepatic stellate cell line LX‐2 (RRID:CVCL_5791), an immortalized human HSC line, was obtained from Sigma‐Aldrich/Millipore (Cat. No. SCC064) within the past 3 years and stored in liquid nitrogen until use. According to the vendor's certificate of analysis, the cells were confirmed to be free of mycoplasma contamination and common viral pathogens and were routinely maintained under aseptic conditions throughout the study. Cells were maintained in T25 or T75 flasks containing Dulbecco's modified Eagle's medium (DMEM; Sigma‐Aldrich) supplemented with 10 IU·mL^−1^ penicillin, 10 IU·mL^−1^ streptomycin, and 2% fetal bovine serum. Cultures were incubated at 37 °C in a humidified atmosphere of 5% CO_2_.

Primary mouse HSCs were isolated from 24‐week C57BL/6 wild‐type mice by *in situ* collagenase perfusion. Briefly, mice were anesthetized, sprayed with 70% ethanol, and laparotomized to expose the portal vein. Following cannulation with a 27G needle, the liver was perfused at 5 mL·min^−1^ with EGTA solution for 5 min, during which the inferior vena cava was incised for blood drainage. Perfusion was then continued with type I collagenase solution at the same rate for 5 min. The liver was excised, the gallbladder removed, and gently dissociated in collagenase solution under sterile conditions. The suspension was filtered through a 70‐μm cell strainer and washed with low‐glucose DMEM. After centrifugation (300 rpm, 2 min, 28 °C, ~100 ***g***), the supernatant containing nonparenchymal cells was collected and sequentially filtered and centrifuged. The resulting pellet was resuspended in primary mouse HSC medium (high‐glucose DMEM supplemented with 10 μm vitamin A, 50 ng·mL^−1^ insulin, and 1% penicillin/streptomycin), and viable cells were counted by trypan blue exclusion. Cells were seeded onto collagen‐coated plates at the desired density, allowed to adhere for 4 h at 37 °C in 5% CO_2_, after which the medium was replaced to remove contaminating hepatocytes. Experiments were conducted within 2–3 days of culture to maintain cell phenotype. This procedure was performed with modifications based on the previously described method [12, 13].

### Vesicular MANT‐ATP accumulation analysis

Cells were seeded into four‐well, 35‐mm glass‐bottom dishes at a density of 2.0 × 10^4^ cells per well. On the following day, the culture medium was replaced, and the cells were preincubated for 3 h with or without clodronate (10 μm, Clo; Tokyo Chemical Industry Co., Ltd. (TCI), Tokyo, Japan) or imeglimin (10 μm, IMG; MedChemExpress LLC, Monmouth Junction, NJ, USA). Subsequently, 2′/3′‐O‐(N‐methylanthraniloyl)‐ATP (MANT‐ATP; Thermo Fisher Scientific) was added to a final concentration of 5 mm, and the cells were incubated for an additional 8 h. Fluorescence imaging was then performed using a STELLARIS 5 confocal microscope (Leica Microsystems GmbH, Wetzlar, Germany).

To assess vesicular MANT‐ATP accumulation, fluorescence intensity was quantified at the individual vesicle level. Corrected vesicular fluorescence (CVF) was defined as the integrated density of each vesicle after background subtraction. The total CVF per cell was calculated by summing the CVF values of all vesicles within a given cell. Three experimental groups were analyzed, with 5–8 cells per group. All image analyses were performed using the Fiji imagej software (version 1.54f).

### ATP release assay of LX‐2 cells

LX‐2 cells were seeded in 24‐well collagen‐coated plates at a density of 5.0 × 10^4^ cells per well. After 2 days, cells were washed and preincubated for 18 h in growth medium containing either IMG (10 μm) or Clo (10 μm), or vehicle as control. Following preincubation, cells were stimulated with ionomycin (Cayman, 5 μm) for 20 min, and half of the supernatant from each well was collected. In a separate set of experiments designed to mimic fibrotic conditions, recombinant human transforming growth factor‐β1 (TGF‐β1; 240‐B, R&D systems, Minneapolis, MN, USA, 5 ng·mL^−1^) was added after drug incubation, and cells were further incubated for 6 h. ATP concentrations in the collected culture supernatants were measured using the ATP Bioluminescence Assay Kit CLS II (11699695001; Roche) according to the manufacturer's protocol. Luminescence was detected using a microplate reader (Infinite 200; Tecan Group Ltd., Männedorf, Switzerland).

### Statistical analysis

All data are presented as mean ± standard error of the mean (SEM). Statistical analyses were conducted using graphpad Prism version 10.4.0 (graphpad Software). Comparisons between two groups were performed using an unpaired two‐tailed Student's *t*‐test. For comparisons involving three or more groups, one‐way analysis of variance (ANOVA) followed by Tukey's or Dunnett's *post hoc* test was employed, as appropriate. A *p* value of < 0.05 was considered statistically significant.

## Results

### Imeglimin ameliorates thioacetamide‐induced body weight loss and liver inflammation

**Fig. 1:**
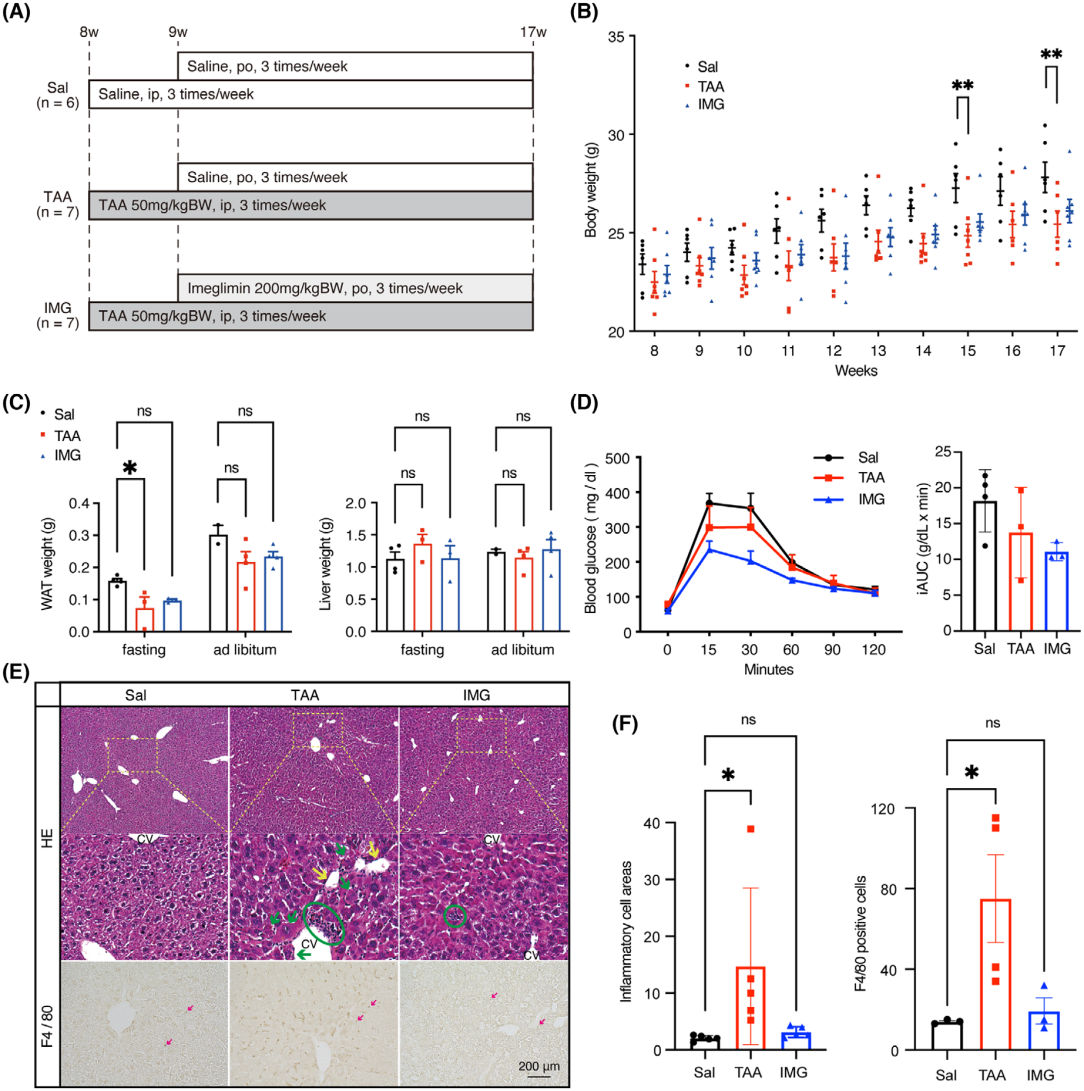
Imeglimin ameliorates thioacetamide‐induced body weight loss and liver inflammation in C57BL/6J mice. (A) Schematic representation of the experimental protocol illustrating the treatment regimen for different groups of C57BL/6J mice subjected to saline or thioacetamide followed by oral administration of saline or imeglimin. (B) Time‐course analysis of body weight changes in mice post‐thioacetamide treatment. (C) Measurement of white adipose tissue (WAT) and liver weights at the end of the intervention period, presented separately for mice sacrificed under fasting (*n* = 4 for Sal; *n* = 3 for TAA; *n* = 3 for IMG) or *ad libitum* conditions (*n* = 2 for Sal; *n* = 4 for TAA; *n* = 4 for IMG). (D) Intraperitoneal glucose tolerance test (ipGTT) conducted at 12 weeks of age. The area under the curve (AUC) is depicted in the right panel (*n* = 6 for Sal; *n* = 7 for TAA; *n* = 7 for IMG). (E) Representative micrographs of hematoxylin and eosin (HE) staining and F4/80 immunostaining of liver sections postintervention (*n* = 6 for Sal; *n* = 7 for TAA; *n* = 7 for IMG). Enlarged views (middle panel) highlight yellow dashed rectangle areas. Inflammatory cell clusters are denoted by green circles, while large necroptotic hepatocytes surrounded by inflammatory cells are indicated by green arrows. Yellow arrows point to blank areas surrounded by inflammatory cells. Red arrows indicate F4/80‐positive macrophages. (F) Quantitative analysis of inflammatory cell infiltration area and F4/80‐positive cell numbers. Inflammatory cell areas were quantified as the mean of five high‐powered fields per animal (*n* = 5 per group). F4/80 positive cells were quantified as the mean of five high‐powered fields per animal (*n* = 3 for Sal; *n* = 4 for TAA; *n* = 3 for IMG). Scale bar = 200 μm. Data are expressed as mean ± SEM. Statistical significance was determined by one‐way ANOVA followed by Tukey's *post hoc* test and indicated as follows: *P < 0.05, **P < 0.01. CV, central vein; IMG, imeglimin; i.p., intraperitoneal injection; p.o., per os (oral administration); Sal, saline; TAA, thioacetamide; WAT, white adipose tissue.

We evaluated whether imeglimin has a protective effect against liver fibrosis *in vivo*. Eight‐week‐old C57BL/6 male mice were administered 50 mg·kg^−1^ thioacetamide (TAA), a hepatotoxicant to induce liver fibrosis, intraperitoneally (i.p.) three times a week for nine consecutive weeks to induce liver fibrosis. Administration of either the vehicle or 200 mg·kg^−1^ imeglimin (IMG) was initiated 1 week later (Fig. 1A). Throughout the intervention period, the TAA‐treated group exhibited a progressive decline in body weight and ultimately weighed significantly less than the normal control (saline only, Sal) group. Coadministration of imeglimin (TAA + IMG) partially attenuated this weight loss, implying a protective effect of imeglimin against TAA‐induced liver injury (Fig. 1B). At the end of intervention, TAA administration tended to decrease white adipose tissue mass, whereas concomitant imeglimin treatment mitigated this reduction. Liver weight remained unchanged across all groups (Fig. 1C). Intraperitoneal glucose tolerance test (ipGTT) at Week 16 showed a trend toward improved glucose tolerance in the imeglimin treatment group compared to the Sal or TAA group, suggesting that imeglimin administration may have exerted a clinically meaningful effect (Fig. 1D). After 9 weeks of TAA exposure (8 weeks of concomitant imeglimin administration), imeglimin markedly attenuated hepatic inflammation on histological examination. H&E‐stained sections from TAA‐treated mice exhibited extensive hepatocellular necrosis and dense inflammatory infiltrates relative to controls, whereas these lesions were substantially mitigated in the IMG group (Fig. 1E,F). Consistent with these findings, immunostaining for the macrophage marker F4/80 revealed abundant F4/80‐positive cell infiltration in TAA livers, which was largely absent following imeglimin treatment.

**Fig. 2:**
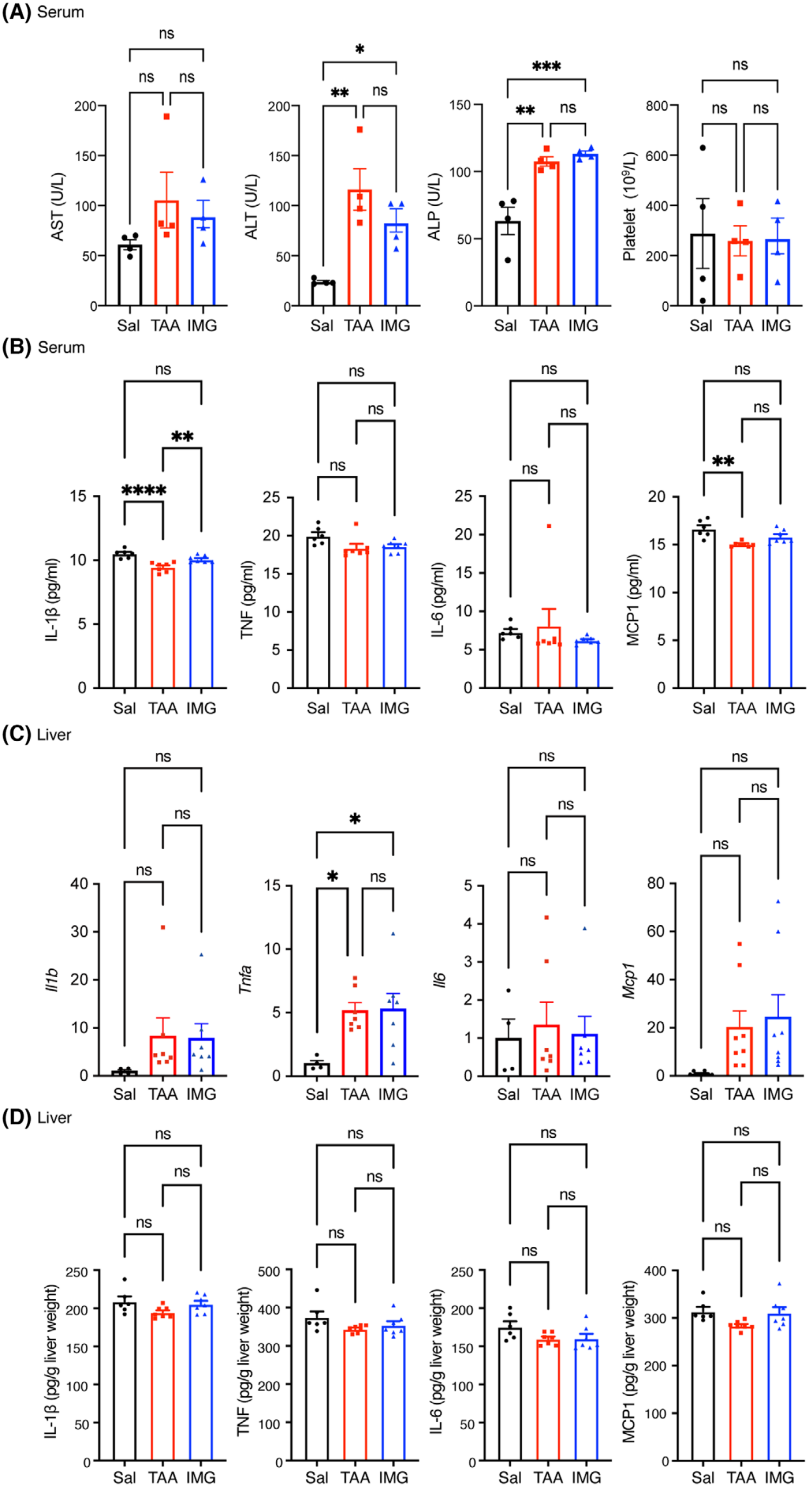
Effect of Imeglimin on serum and hepatic inflammatory mediators in C57BL/6J mice exposed to thioacetamide (TAA). (A) Serum activities of AST, ALT, and ALP measured using the Vetscan VS2 chemistry analyzer and blood platelet counts measured using the Vetscan HM5 hematology analyzer (*n* = 4). (B) Serum concentrations of IL‐1β, TNF, IL‐6, and MCP1 assessed using BD CBA (*n* = 6 for Sal; *n* = 7 for the intervention groups). (C) Hepatic mRNA expression of *Il1b*, *Tnfa*, *Il6*, and *Mcp1* quantified by quantitative real‐time PCR (qRT‐PCR) (*n* = 4 for Sal; *n* = 7 for the intervention groups). (D) Tissue levels of IL‐1β, TNF, IL‐6, and MCP‐1 in liver homogenates assessed by BD CBA (*n* = 6 for Sal; *n* = 7 for the intervention groups). Data presented as mean ± SEM. Statistical significance was determined by one‐way ANOVA followed by Tukey's *post hoc* test and indicated as follows: *P < 0.05, **P < 0.01, ***P < 0.001, ****P < 0.0001. ALT, alanine aminotransferase; ALP, alkaline phosphatase; AST, aspartate aminotransferase; BD CBA, BD® Cytometric Bead Array; IL, interleukin; IMG, imeglimin; MCP1, monocyte chemoattractant protein‐1; Sal, saline; TAA, thioacetamide; TNF, tumor necrosis factor.

At the end of the study, serum alanine aminotransferase (ALT) and alkaline phosphatase (ALP) activities were elevated in both TAA‐treated groups, irrespective of imeglimin treatment, indicating persistent hepatocellular injury. Platelet counts—which typically decline as fibrosis progresses—remained unchanged across groups (Fig. 2A).

Quantitative profiling of circulating inflammatory cytokines revealed no biologically meaningful differences among the three groups; absolute serum concentrations of IL‐1β, TNF, IL‐6, and MCP‐1 were comparable despite minor statistical fluctuations (Fig. 2B).

Consistently, hepatic mRNA expression of *Il1b*, *Il6*, and *Mcp1* did not differ between groups, whereas *Tnfa* transcripts were significantly upregulated in TAA‐exposed mice (Fig. 2C). Nevertheless, hepatic protein levels of IL‐1β, TNF, IL‐6, and MCP‐1, determined by Cytometric Bead Array, were essentially equivalent among Sal, TAA, and IMG groups (Fig. 2D).

### Imeglimin ameliorates HSC activation and improves hepatic fibrosis

**Fig. 3:**
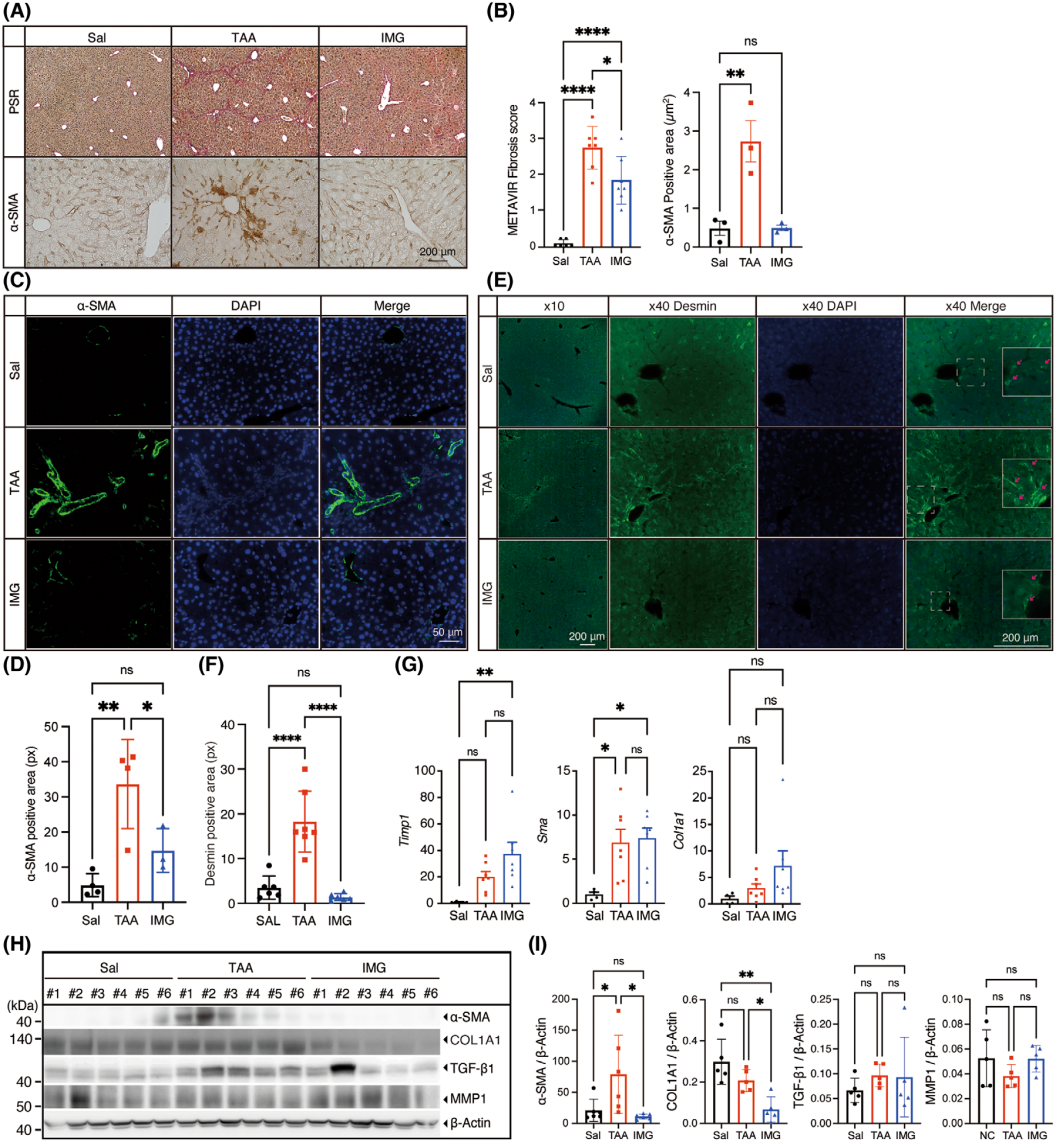
Imeglimin ameliorates thioacetamide‐induced liver fibrosis in C57BL/6J mice. (A, B) Representative micrographs depicting liver sections stained with Picro‐Sirius Red (PSR) and α‐Smooth Muscle Actin (α‐SMA) following intervention and quantitative analysis of METAVIR fibrosis scores (*n* = 6 for Sal; *n* = 7 for the intervention groups) and α‐SMA‐positive area (*n* = 3 for each group) as the mean of five high‐powered fields per animal. Scale bar = 200 μm. (C, D) Fluorescence micrographs displaying α‐SMA staining of liver sections postintervention and quantitative analysis of α‐SMA‐positive area (*n* = 4 for Sal; *n* = 4 for TAA; *n* = 3 for IMG). Nuclei stained with DAPI (blue). Scale bar = 50 μm. (E, F) Fluorescence micrographs of Desmin staining. Red arrows indicate the stellate shape of HSCs. (F) Quantitative analysis of Desmin positive area. (*n* = 6 for Sal; *n* = 7 for TAA; *n* = 6 for IMG). (G) Real‐time PCR (RT‐PCR) analysis of fibrosis marker genes *Timp1, Sma, and Col1a1* in liver tissue. (*n* = 4 for Sal; *n* = 7 for the intervention groups). (H, I) Western blot analysis and densitometric quantification of α‐SMA, COL1A1, TGF‐β1, and MMP1 in mouse livers. β‐Actin used as an internal control. Biological replicate samples labeled as #1–#6. Data presented as mean ± SEM. Statistical significance was determined by one‐way ANOVA followed by Tukey's *post hoc* test and indicated as follows: *P < 0.05, **P < 0.01, ****P < 0.0001. *Col1a1*, *collagen type I alpha 1*; DAPI, 4′,6‐diamidino‐2‐phenylindole; IMG, imeglimin; MMP1, matrix metalloproteinase 1; Sal, saline; TAA, thioacetamide; TGF‐β1, transforming growth factor beta 1; *Timp1*, *tissue inhibitor of metalloproteinases 1*.

Next, we evaluated fibrosis as a downstream consequence of chronic hepatic inflammation. Collagen deposition was visualized with PSR staining and semi‐quantified using the METAVIR scoring system; imeglimin reduced the fibrosis stage by one grade relative to the TAA group (Fig. 3A,B). Activation of HSC (the principal collagen‐producing cells in fibrogenesis) was assessed by DAB immunostaining for the activation marker α‐SMA. Quantification of the stained area revealed a marked attenuation of stellate‐cell activation in mice receiving imeglimin (Fig. 3A,B). Fluorescence immunohistochemistry corroborated these findings (Fig. 3C,D). To further confirm the activation of HSCs by TAA and its attenuation by imeglimin treatment, we performed immunohistochemistry for desmin, a marker of HSCs. Although desmin is expressed in both quiescent and activated HSCs, its expression is stronger in the latter. As shown in Fig. 3E, proliferating HSCs were predominantly observed in pericentral venous areas of the TAA group, whereas comparable quiescent levels were observed in the Sal and IMG groups. This finding was further supported by quantitative analysis (Fig. 3F).

Although the transcription of α‐SMA in liver lysates from the IMG group, as assessed by RT‐qPCR, was comparable to that observed in the TAA group (Fig. 3G), immunoblot analysis further confirmed that imeglimin attenuated the TAA‐induced increase in α‐SMA protein levels (Fig. 3H,I). Furthermore, while Col1A1 transcription—a marker of collagen synthesis—tended to be higher in both the TAA and IMG groups compared to the Sal group, and Timp1 transcription—a gene involved in extracellular matrix maintenance—was significantly elevated in the IMG group relative to the TAA and Sal groups (Fig. 3G), imeglimin reduced COL1A1 protein abundance. In contrast, the expression levels of the profibrogenic cytokine transforming growth factor‐β1 (TGF‐β1) and matrix metalloproteinase 1 (MMP1) remained unaltered (Fig. 3H,I).

### Imeglimin attenuates VNUT dependent ATP secretion by HSCs

Collectively, the murine studies demonstrated that imeglimin attenuates inflammatory‐cell infiltration, HSC activation, and collagen deposition in the TAA model. Because serum ALT and ALP activities were elevated to a similar extent in the TAA and IMG groups, the compound is unlikely to confer direct hepatocellular protection against TAA toxicity; rather, its antifibrotic effect appears to stem from interference with downstream inflammatory and HSC‐mediated events. These findings prompted us to examine adenosine triphosphate (ATP), a representative damage‐associated molecular pattern (DAMP), as a potential mechanistic target.

Imeglimin was derived from metformin [14], and Senfeld et al. showed that metformin dampens purinergic signaling by inhibiting vesicular ATP release [15]. Notably, their experiments employed physiologically attainable concentrations of 10–30 μm, in contrast to earlier work that used supraphysiological doses. We recently found that murine HSCs express the vesicular nucleotide transporter (VNUT), actively package ATP into secretory vesicles, and release it to engage purinergic pathways. Furthermore, we discovered that clodronate, a VNUT‐specific inhibitor, exerts an antifibrotic effect in the TAA mouse model, thereby confirming that vesicular ATP storage and release are involved in HSC activation and fibrogenesis (manuscript submitted and under review). Accordingly, we hypothesized that clinically relevant concentrations of imeglimin might suppress vesicular ATP loading and, consequently, purinergic signaling, thereby exerting anti‐inflammatory and antifibrotic effects.

**Fig. 4:**
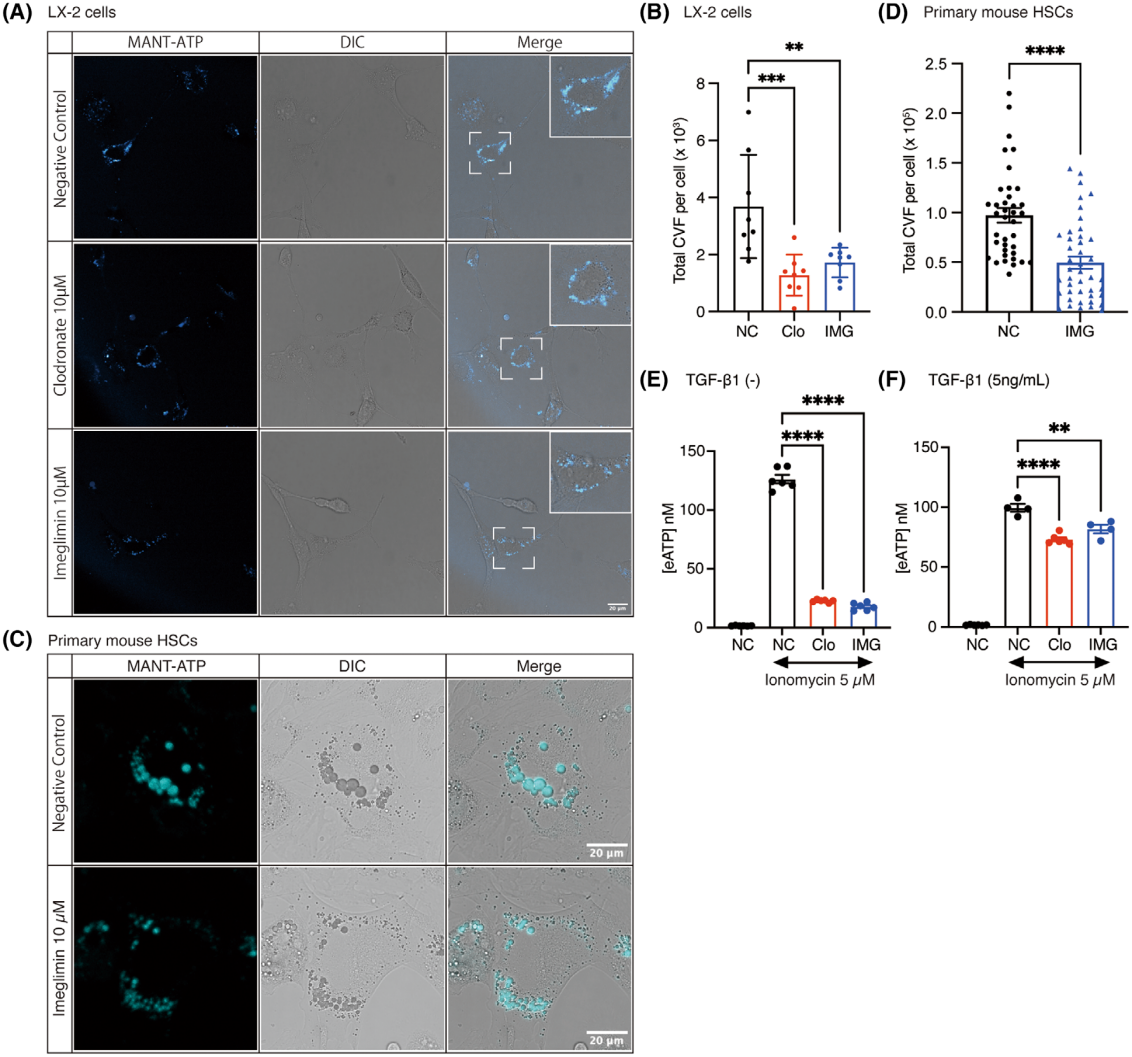
Impact of imeglimin on ATP accumulation and ATP secretion in LX‐2 cells. (A, B) Live‐cell imaging illustrating MANT‐ATP fluorescence within ATP‐containing secretory vesicles. Clodronate served as a positive control, known to hinder ATP accumulation. Scale bar = 20 μm. (B) Quantification of vesicular MANT‐ATP accumulation as total intracellular corrected vesicular fluorescence (CVF) (*n* = 8 from two independent experiments). (C, D) Live‐cell imaging illustrating MANT‐ATP fluorescence within ATP‐containing secretory vesicles. Scale bar = 20 μm. (D) Quantification of vesicular MANT‐ATP accumulation as total intracellular CVF (*n* = 38 for NC, *n* = 43 from two independent experiments). (E, F) Assessment of ATP secretion in LX‐2 cells upon exposure to the calcium ionophore ionomycin at a concentration of 5 μm after 18 h of drug pre‐incubation. (*n* = 6) f. with 12 h of TGF‐β1 stimulation at a concentration of 5 ng·mL^−1^ (*n* = 6 for NC, Clo; *n* = 4 for NC with Ionomycin and IMG). Data shown are from a single representative experiment, which was repeated three times with comparable outcomes. Data shown as mean ± SEM. Statistical significance was determined by one‐way ANOVA followed by Tukey's *post hoc* test (B, E, F) and by unpaired two‐tailed Student's *t*‐test and indicated as follows: denoted as **P < 0.01, ***P < 0.001, ****P < 0.0001. Clo, clodronate; DIC, differential interference contrast; IMG, imeglimin; NC, negative control.

To test this hypothesis, we first performed a MANT‐ATP uptake assay in the human HSC line LX‐2 and primary mouse HSCs. The fluorescent ATP analogue MANT‐ATP is a validated probe for monitoring vesicular ATP accumulation [16]. An 18‐h incubation with 10 μm imeglimin reduced intravesicular fluorescence to a level comparable to that observed with 10 μm clodronate, a known VNUT inhibitor, suggesting impaired ATP uptake in LX‐2 cells (Fig. 4A). Quantification of total vesicular fluorescence within cells confirmed this finding and demonstrated a significant reduction in MANT‐ATP vesicular accumulation following treatment with 10 μm imeglimin, to an extent similar to that induced by 10 μm clodronate (Fig. 4B). Consistent with this result, vesicular ATP content was diminished in imeglimin‐treated LX‐2 cells.

With primary HSCs, we also confirmed the inhibitory effect of imeglimin on vesicular ATP storage (Fig. 4C,D). However, because primary HSCs were obtained from crude mesenchymal cell preparations that included not only HSCs but also fibroblasts, monocytes, and endothelial cells, the resulting cultures were not completely pure, even when maintained in vitamin A‐containing medium that preferentially supports HSC growth. For this reason, we employed LX‐2 cells, which represent a pure HSC population, to assess vesicular ATP release and the effect of imeglimin. Following ionomycin‐induced exocytosis, extracellular ATP concentrations were likewise decreased, mirroring the effect of clodronate (Fig. 4E). Finally, when LX‐2 cells were pretreated with imeglimin and stimulated with 5 ng·mL^−1^ TGF‐β1 to mimic the fibrotic milieu, both vesicular ATP storage and ionomycin‐evoked release remained suppressed (Fig. 4F).

Taken together, these data suggest that imeglimin limits vesicular ATP loading and secretion in HSCs, thereby blunting purinergic signaling in stellate cells—and potentially in infiltrating immune cells—which may underlie the compound's anti‐inflammatory and antifibrotic activities observed *in vivo*.

## Discussion

In the nine‐week TAA model, imeglimin markedly reduced hepatic inflammatory‐cell infiltration, lowered histological fibrosis scores, and attenuated HSC activation, while serum ALT and ALP rise remained unchanged. Although profound reductions in infiltrating immune cells were observed, neither systemic nor intrahepatic concentrations of canonical cytokines decreased. This finding is consistent with previous reports that cytokine levels in the TAA model fluctuate over time and may plateau during chronic exposure [17, 18]. Together, these results suggest the involvement of alternative chemoattractants.

A previous study on metformin—the parent compound of imeglimin—showed that it inhibits vesicular ATP release and purinergic signaling, thereby enhancing insulin signaling and improving insulin resistance in hepatocytes [13]. We have recently shown (manuscript submitted and under review) that LX‐2 cells express VNUT and multiple P2X receptors—including P2X purinoceptor 4, P2X5, P2X7, and P2X10. In addition, the pharmacological inhibition of VNUT in LX‐2 cells by clodronate suppressed cell proliferation and collagen deposition. Our data in this article extend these findings by demonstrating a direct action of imeglimin on HSC activation and by documenting vesicular ATP release followed by purinergic signaling as its plausible target. The translational relevance of these findings is supported by pharmacological consistency with human therapy. *In vivo*, mice received a dose calculated using the standard human‐to‐mouse allometric conversion (200 mg·kg^−1^·day^−1^), which replicates therapeutic exposure. *In vitro* experiments were conducted at a concentration of 10 μm, corresponding to the reported peak plasma levels in patients (approximately 11.4 μm; equivalent to 2.2 μg·mL^−1^ of imeglimin hydrochloride, the commercial form of imeglimin, with a molecular weight of 191.66 g·mol^−1^) [19]. Previous studies reporting the anti‐inflammatory properties of imeglimin have employed higher concentrations. For instance, Kato et al. demonstrated anti‐inflammatory effects in high‐glucose‐stimulated mouse microglia using a concentration of 500 μm [20], while Lee et al. observed attenuation of proinflammatory cytokine expression in THP‐1 macrophages at 50 μm and 100 μm [9]. Notably, to our knowledge, this is the first report demonstrating pharmacodynamic effects of imeglimin at clinically relevant concentrations as low as 10 μm *in vitro*, thereby reinforcing the translational validity of our findings.

These considerations support prioritizing imeglimin for individuals with type 2 diabetes who are at increased risk of liver injury, such as those with obesity or confirmed hepatic steatosis. Moreover, imeglimin may hold promise as a potential antifibrotic agent across a range of etiologies—including viral hepatitis and toxin‐induced liver injury—without necessitating supra‐therapeutic dosing.

The TAA model represents toxin‐mediated necro‐inflammation; its relevance to metabolic or autoimmune fibrosis requires confirmation in dietary and genetic models. Previous studies utilizing choline‐deficient‐fat‐induced murine MASLD model have linked the hepatoprotection and antifibrotic effect of imeglimin to improvements in hepatocellular lipid handling [21]. Taken together, current evidence supports a dual mechanism whereby imeglimin exerts antifibrotic effects via (i) metabolic reprogramming of hepatocytes and (ii) direct inhibition of HSC activation.

Several limitations should be acknowledged. Imeglimin is already approved for the treatment of type 2 diabetes and has demonstrated a favorable safety profile in clinical settings, with gastrointestinal symptoms being the most reported adverse effects and no evidence of serious complications such as lactic acidosis. Notably, the present study demonstrated antifibrotic effects at doses equivalent to those used in diabetes therapy. However, the long‐term safety and efficacy of imeglimin for liver fibrosis specifically—particularly with chronic administration—remain to be fully characterized. Additionally, its potential to reverse established cirrhosis has yet to be determined.

Future investigations should clarify time‐dependent efficacy (prevention versus reversal), delineate the contribution of individual P2X receptors to imeglimin actions, and explore combination therapy with GLP‐1 receptor agonists or emerging antifibrotic compounds. Prospective clinical trials incorporating noninvasive fibrosis biomarkers and pharmacokinetic readouts will be essential to translate these preclinical insights into personalized liver care.

Collectively, our results position imeglimin as a dual‐action agent that corrects metabolic derangements while directly interrupting purinergic signaling in HSCs, offering a promising therapeutic avenue for both metabolic and nonmetabolic liver fibrosis.

## Author Contributions

**Seiji Nomura:** investigation, visualization, and writing – original draft. **Lixiang Wang:** conceptualization, investigation, visualization, writing – review and editing, and funding acquisition. **Nao Hasuzawa:** conceptualization, methodology, project administration, and funding acquisition. **Ayako Nagayama:** supervision, funding acquisition. **Sawako Moriyama:** investigation, methodology, and validation. **Kenji Ashida:** writing – review and editing, supervision, and funding acquisition. **Yoshinori Moriyama:** conceptualization, supervision, and funding acquisition. **Masatoshi Nomura:** supervision, funding acquisition. **Ken Yamamoto:** supervision, funding acquisition.

## Conflict of interest

The authors declare no conflict of interest.

## Data Availability

The datasets supporting the conclusions of this study are available from the corresponding author upon reasonable request.
